# Evolving national dementia policies in the OECD: Prevention, diagnosis, and care

**DOI:** 10.1002/alz.71367

**Published:** 2026-06-01

**Authors:** Soohyun Kim, Judit Rauet‐Tejeda, Ricarda Milstein, Jinkook Lee, Ana Llena‐Nozal

**Affiliations:** ^1^ Organisation for Economic Co‐operation and Development Paris France; ^2^ Department of Economics and Center for Economic and Social Research University of Southern California Los Angeles California USA

**Keywords:** dementia, dementia care pathways, dementia diagnosis, national dementia strategies, Organisation for Economic Co‐operation and Development, policy analysis

## Abstract

Dementia incurs yearly costs of nearly 1.3 trillion US dollars worldwide, projected to rise significantly in the coming decades due to population aging. This policy review provides an overview of the latest national dementia strategies, diagnostic guidelines, and treatment guidelines across 38 Organisation for Economic Co‐operation and Development countries. Our findings suggest that national dementia policies increasingly emphasize prevention through modifiable risk factors, early diagnosis, and non‐pharmacological treatment approaches, progressing toward more coordinated care pathways. Clinical guidelines have evolved to incorporate new research evidence and practices in dementia diagnostics and treatment over the past decade. Despite updates to policy frameworks, we identified a gap between policy and action, with varying degrees of implementation and support for improving diagnosis and adopting new treatments. We identified a series of policy efforts that could be undertaken to enhance the quality of care for people living with dementia.

## 1 INTRODUCTION

Dementia is one of the leading causes of death in old age across the Organisation for Economic Co‐operation and Development (OECD), and with ongoing population aging, the number of people living with dementia is estimated to increase from 21 million in 2021 to almost 32 million by 2040 across OECD countries.^1^ Dementia care leads to substantial costs, ranging from EUR 32,6076.9 to 162.9 million per annum in Europe^2^ and USD 781 billion in the US in 2025,^3^ including medical and long‐term care costs along with the personal economic toll.

Creating and implementing national dementia plans and strategies is a crucial policy lever for improving dementia recognition and management. By 2018, at least 22 OECD countries had adopted national dementia strategies focusing on improving diagnosis, post‐diagnostic support, community‐based initiatives, support for informal caregivers, and data collection and measurement.^4^ Over the past decade, many national strategies have been revised or introduced anew in response to the growing impact of dementia on population health. Preventive approaches, raising awareness, and destigmatization of dementia have gained more emphasis, with early diagnosis and other areas as continued priorities. Likewise, clinical guidelines for dementia diagnosis and treatment have also undergone major updates across many countries, reflecting accumulated research evidence and advances in clinical practice.

In this article, we review the latest dementia care policies across OECD countries by analyzing national dementia plans or strategies, diagnostic guidelines, and treatment guidelines. We focus on OECD countries to ensure the comparability and relevance of the findings across similar systems, while keeping the review within a manageable scope. With other comparative studies focusing on specific regions,^5^, ^6^ two studies^7^, ^8^ examined how the inequality agenda is incorporated into OECD countries’ national dementia strategies. However, the results of these studies were largely thematic and limited to national strategies written in English or French. Our work provides a more comprehensive overview of the most recent national strategies and clinical guidelines published in the national languages of all OECD member countries.

## 2 METHODS

We collected qualitative data primarily in two phases: through structured interviews with country experts and follow‐up desk research. Participants in the interviews were recruited through snowball sampling, with referrals made by the OECD country delegations, between June 2024 and November 2024. As a result, we interviewed government officials, experts from relevant public health agencies, heads of dementia care units in regional hospitals, and professors from 29 OECD countries who agreed to participate in structured interviews, either via virtual meetings or in writing, from July 2024 to January 2025. All respondents were informed of the confidentiality of their responses before the interview, and each interview lasted ≈ 1 hour. Both interviews and written questionnaires used the same set of questions, covering the latest dementia policy frameworks and recent changes in dementia diagnosis and care pathways in each country since 2018, when the OECD published its last report on dementia care.

After the data collection, we conducted a thematic analysis to complement the interview findings and better understand the latest policy context by reviewing online materials and publications from governments, international organizations, and dementia‐specific organizations, as well as academic literature.^9^ Specifically, national dementia strategies include dementia‐specific strategies, action plans, policies, or broader aging or brain health frameworks with a dementia agenda at national and subnational levels. Diagnostic and treatment guidelines for dementia include clinical guidelines and care pathways published by the national and subnational governments and relevant medical associations. We prioritized the most recent government documents and excluded non‐official documents such as news articles or advocacy reports. Information in all official languages of the countries studied, plus English, where it is not an official language, was included in the analysis.

We clustered country‐level information using the OECD's analytical categories^4^ and analyzed across national strategies, diagnoses, and treatments. For national strategies, countries were first screened for the availability of a national or subnational dementia strategy, then whether it was a stand‐alone plan or part of a broader strategy, such as healthy aging or brain health. We also extracted year and publisher information, when available. Subsequently, the content of each strategy was assessed relative to common themes that recurred across all strategies. The same process was repeated for analyzing clinical guidelines for diagnosis and treatment. A theme was identified as a common theme if it was named in at least 10 different clinical guidelines or strategies. Meanwhile, we collected additional information on diagnostic pathways and country efforts to improve diagnostic rates, including following the analytical framework by OECD,^4^ covering (1) who performs the diagnosis, (2) where the diagnosis takes place, and (3) diagnostic tools available and the one actually used.

## 3 RESULTS

### 3.1 National dementia strategy

#### 3.1.1 Countries continue to prioritize dementia through developing national strategies

Currently, 30 out of 38 OECD countries (78%) have stand‐alone national dementia plans, whereas 3 countries embed dementia‐related objectives within broader policy frameworks, such as long‐term care (Hungary) or neurodegenerative diseases (France; Table 1). In the case of Estonia, although they lack a national policy, they have the Dementia Competence Centre's (DKK) strategic plan, which serves as a planning tool for the DKK day‐to‐day activities and longer term development work. France transitioned from dedicated Alzheimer's disease (AD) plans to a comprehensive neurodegenerative diseases plan in 2013. Eight countries (24%) have more than one dementia framework because they have plans at both the national and regional levels, such as Spain and Portugal, or because they have additional policy or legislative frameworks related to it (Costa Rica, Finland, Italy, Japan, Korea, and Norway); for example, Japan's Basic Act on Dementia (2024), or South Korea's National Responsibility for Dementia (2017). As a result, 43 dementia policies were collected in total. However, Colombia, Latvia, Lithuania, the Slovak Republic, and Türkiye lack a dedicated national dementia plan or a broader strategy that explicitly addresses dementia.

**TABLE 1:** National dementia strategies implemented across 38 OECD countries.

		Policy		Contents				
Countries	National strategy	Stand‐alone	Broader/other	Prevention	Caregivers	R+D	Diagnosis	De‐stigma/awareness
Australia	National Dementia Action Plan 2024‐2034	●		●	●	●	●	●
Austria	Dementia Strategy—Living well with dementia, 2015	●			●	●	●	●
Belgium (Flanders)	Flanders’ dementia plan 2021–2025	●		●	●			●
Canada	A Dementia Strategy for Canada: Together we Aspire, 2019	●		●	●	●	●	●
Chile	National Dementia Plan 2017–2025	●		●		●		●
Costa Rica	National plan for Alzheimer's disease and related dementias: shared effort 2014–2024	●			●	●	●	●
	National Norm for Care of Adults with Cognitive Impairment and Dementia, 2017		●	●	●		●	
Czechia	National Action Plan for Alzheimer's Disease and Related Illnesses 2020–2030	●		●	●	●	●	●
Denmark	A Safe and Dignified Life with Dementia: National Action Plan on Dementia (2017–2025)	●			●	●	●	●
Estonia	Dementia Competence Center's Strategic Plan 2023–2027		●	●	●		●	●
Finland	Finland National Memory Programme 2012–2020	●		●	●	●		●
	Programme: A strong and committed Finland of Prime Minister Petteri Orpo's Government, [Supports Older Peoples Coping at Home], 2024		●	●	●			
	Quality recommendations to guarantee active and functional aging and sustainable services in 2024‐2027		●	●	●			
France	National Strategy for Neurodegenerative Diseases 2024–2028		●			●		
Germany	National Dementia Strategy, 2020–2026	●		●	●	●		●
Greece	Dementia action plan, 2016–2020	●		●	●	●	●	●
Hungary	Strategy for long‐term care 2023–2030		●		●			●
Iceland	National Dementia Action Plan 2020–2025	●		●	●	●	●	●
Ireland	National dementia strategy, 2014	●		●	●	●	●	●
Israel	Addressing Alzheimer's and Other Types of Dementia: Israeli National Strategy, 2013	●			●			●
Italy	National Plan on Dementia, 2014	●		●			●	●
	Italian National Prevention Plan 2020–2025		●	●				
Japan	National Framework for Promotion of Dementia Policies, 2023	●		●	●	●	●	●
	Basic Act on Dementia to Promote an Inclusive Society, 2024	●		●	●	●	●	●
Korea	National Responsibility for Dementia, 2017		●	●	●		●	●
	Fourth General Measures for Overall Dementia Management (2021–2025)	●		●	●	●	●	●
Luxembourg	National Action Plan for Dementia Diseases, 2013	●		●	●		●	●
Mexico	National Plan Dementia Care, 2024	●		●	●	●	●	●
Netherlands	National Dementia Strategy 2021–2030	●		●	●	●	●	●
New Zealand	Dementia Mate Wareware Action Plan 2026–2031	●		●	●	●	●	
Norway	Dementia Plan, 2025	●		●	●	●	●	●
	The National Brain Health Strategy, 2018–2024		●	●	●	●	●	
Poland	National Action Program for Dementia Diseases for 2025–2033	●		●	●	●	●	
Portugal	Dementia Health Strategy (*Despacho* n.° 5988/2018)	●			●	●	●	●
	Regional health plan for dementia Lisbon and Tagus valley region, 2019		●		●		●	●
Slovenia	Dementia Management Strategy, 2023‐2030	●		●	●	●	●	●
Spain	Alzheimer and other dementias plan for 2019–2023	●		●	●	●	●	●
	Alzheimer and other dementias: strategy on neurodegenerative disorders of the national health system, 2022		●	●	●	●	●	
	Comprehensive Plan for Alzheimer's and Other Dementias in Andalusia, 2021		●	●	●	●	●	●
Sweden	Everyday Counts: National Dementia Strategy 2025–2028	●		●	●	●	●	
Switzerland	National Dementia Platform, 2021	●			●	●	●	●
United Kingdom (Scotland)	Dementia strategy: Everyone's Story, 2023	●		●	●	●	●	●
United States	National Plan to Address Alzheimer's Disease, 2024	●		●	●	●	●	●
Total^a^		31	12	34	39	31	33	32

*Note*: The “Policy” column distinguishes between two types of strategies: (1) stand‐alone policies which represent the national dementia policy, and (2) dementia‐related policies that are embedded within broader strategic frameworks, correspond to regional strategies, or are legal acts. The “Contents” columns refer to the most common topics included in these strategies and plans. *Source*: OECD own analysis/questionnaire.

^a^The total number of policies, strategies, and plans is out of 43.

Abbreviations: OECD, Organisation for Economic Co‐operation and Development; R&D, research and development.

Over the past decade, more than half of OECD countries have updated national dementia plans and strategies. While 36% (11/30) have not revised their initial strategy established before 2018, 63% (19/30) of countries have updated their national strategies and six countries that lacked a strategy in 2018 have since introduced it (Czechia, Germany, Iceland, Mexico, and Poland). Czechia, Germany, Greece, and Switzerland are expected to publish new policy documents within 1 to 2 years. The Ministry of Health published these national strategies in ≈ 76% of the strategies examined, sometimes in collaboration with the Ministry of Social Affairs. In other cases, different ministries or health‐related institutions were responsible for releasing the strategies. These include the National Institute of Geriatrics in Mexico, the National Council for Older Persons (CONAPAM) in Costa Rica, the Social Care Ministry in Scotland, and the Ministry for Older People and Social Security in Sweden.

#### 3.1.2 Many countries have similar themes featuring prominently in their policies

We identify that national dementia strategies commonly organize policy measures around five common themes: caregiver support, awareness‐raising/destigmatization, prevention, diagnosis, and research and development (R&D) investment.

Caregiver support is the most frequently included component of national dementia strategies, with 39 strategies (90%) having measures to support and inform dementia caregivers in their roles. In some countries, strategies include more targeted action plans to improve caregivers’ well‐being. In the US, supporting caregivers is a key focus of its national dementia strategy, particularly its third goal, which outlines detailed strategies.^10^ Other countries provide more general, non–dementia‐specific support for caregivers, such as travel vouchers, leisure services, counseling services, and workplace policies. Finland provides various supports for informal caregivers, which can also benefit dementia caregivers, including service vouchers, home care, and respite options. Korea offers online psychological self‐screening and counseling, and outbound case management through the National Dementia Helpline for family caregivers, and Germany also provides counseling services. Workplace policies that support caregivers in balancing work and caregiving (e.g., part‐time, remote work, and caregivers’ leave) are also available. Furthermore, Germany aims to strengthen new forms of cooperation through local networks of care and support services.

Thirty‐two out of 43 strategies (74%) include actions to raise awareness and reduce stigma around dementia. In Germany, the federal government is launching a national awareness campaign and promoting “Dementia Friends” training across various occupational groups to help build a dementia‐friendly environment. Efforts also target children and youth to increase understanding of the condition. Similarly, Japan's Basic Act on Dementia highlights the importance of promoting public understanding to foster a more inclusive society by ensuring that people have accurate knowledge about dementia. This includes promoting dementia awareness through education and supporting people with dementia in sharing their experiences, for example, through Dementia Hope Ambassadors.

Thirty‐four out of 43 dementia strategies (79%) explicitly include measures for preventing dementia as a key component. Countries aim to prevent dementia and increase early detection through different strategies. A common approach involves extensive public health campaigns promoting healthy living, as seen in the plans from Germany, Canada, Iceland, and Norway. Other actions aim to prevent dementia through educating the public, as seen in Sweden, where the Dementia Centre offers free training on lifestyle changes for brain health, and in Australia, where Dementia Australia provides guidance on exercise, heart health, mental stimulation, and social engagement. A step forward in this context are prevention measures that address multiple lifestyle factors at once.

Seventy‐seven percent of strategies include measures to improve dementia diagnosis, with countries like Australia and Mexico adopting approaches tailored to their specific needs. Both countries share a commitment to improving early diagnosis, coordinated care, and training for health‐care providers. Australia's approach includes broader system reforms, regular reviews and updates of clinical practice guidelines, clear national pathways for screening and diagnosis, and reassessment of funding models for memory clinics. In addition, it includes embedding memory clinics within Aboriginal Community Controlled Health Organisations to improve access and ensure culturally appropriate care for First Nations people. Mexico's National Dementia Plan focuses on improving dementia diagnosis and management within health‐care institutions. The strategy emphasizes co‐ordinating care across all levels to optimize diagnosis and follow‐up, reorganizing existing services instead of creating new ones to prevent overburdening the system.

Lastly, R&D, including data collection efforts, was incorporated into 72% of dementia strategies, indicating an increasing investment in innovation to advance dementia research and clinical pathways. Norway's Brain Health Strategy includes a government allocation of NOK 20 million through the Norwegian Research Council to establish new clinical research centers for serious central nervous system diseases, including dementia. In its previous national dementia strategy (2014–2019), Switzerland also set out specific objectives focused on improving data collection on current and future dementia care at the cantonal level and enhancing the exchange between researchers and care providers.

### 3.2 Diagnosis

#### 3.2.1 In most OECD countries, general practitioners give the initial diagnosis, which is confirmed by a specialist

General practitioners (GPs) are the first point of contact for people with dementia in most countries (Table S1 in supporting information). Across all OECD countries (for which information is available), there has been no change in the past decade. The role of primary care in dementia detection is often formalized or highlighted in national dementia plans and is often linked in those plans with goals to promote timelier diagnosis. In nearly all countries, GPs perform at least a basic cognitive assessment and provide people with an indicative diagnosis.

A formal diagnosis is often made by specialists but a referral from a GP is usually required. Generally, suspected dementia cases first go to a GP for a basic cognitive assessment to detect dementia before being referred to specialists for a more comprehensive assessment. It is found that in all countries the formal diagnosis is performed by a specialist, which varies by country (e.g., neurologists, geriatricians, psychiatrists, and neuropsychologists). In many countries, a specialist's formal diagnosis is necessary for full or partial reimbursement of social and clinical support services, including medications and home care. However, for a limited number of countries, primary care physicians and sometimes even non‐physician health professionals are also allowed to initiate and follow up on the prescription of medications to relieve the symptoms of dementia. In Australia, Denmark, Korea, Mexico, Sweden, and the UK, GPs are allowed to prescribe medications, while in Spain they can follow up but not initiate it.^11^, ^12^ In the Netherlands, nurse practitioners are also allowed to start prescribing medications for people with dementia.^13^

In addition, nurses also support the role of GPs, for example by assisting with early identification of suspected dementia cases or facilitating referrals. Nurses sometimes are part of the teams working in memory clinics in Israel, Greece, England (UK), and Finland, hence, are involved in the diagnosis process. In Australia, Canada, Denmark, Ireland, and Sweden, nurses support GPs in primary care settings. A systematic review highlights potential benefits such as improved patient access, earlier detection and management of cognitive decline, and enhanced care coordination. However, some limitations discussed are unclear role definition, insufficient dementia‐specific training, time pressures, and weak communication with GPs.^14^

While the GPs are the most common contact point in some countries, patients can directly go to a specialist. In Germany, Greece, Ireland, Japan, Korea, and Latvia, an initial consultation with primary care practitioners is not necessary for dementia diagnosis. In Germany, people can directly access a specialist when symptoms of dementia are present. The Japanese health system does not register GPs in a separate category from specialists, so insured patients are allowed to go directly to any physician.^15^, ^16^ In Greece, Korea, and Latvia, patients can bypass the primary care system in favor of going directly to a neurologist or other specialist. In Ireland, older people can enter the specialist diagnostic pathway through referrals from psychiatrists or neurologists at regional memory clinics.

In some countries, GPs can perform a formal diagnosis. In Korea, at medical institutions (including hospitals affiliated with dementia care centers), GPs, family medicine specialists, neurologists, and psychiatrists can provide consultations, index diagnosis, formal diagnosis (diagnostic tests), and drug treatments.

The typical settings where diagnoses take place are primary care facilities and hospitals. However, in some countries, such as Iceland, Ireland, and Finland, memory clinics are responsible for the formal diagnosis. These memory clinics are defined broadly as clinical settings where health professionals deliver advanced diagnostic services. Some memory clinics may focus solely on diagnosis or go beyond it to provide ongoing care and support. In Greece, a dementia diagnosis can take place in memory clinics or day care centers. In Finland, it can occur in home care and 24/7 care centers, while in Japan and Korea in memory clinics which are labeled Dementia Medical centers. In another group of countries (Israel, Italy, and Latvia), the range of settings broadly includes primary health care, long‐term care institutions, or through professionals providing long‐term care at home, disability services, and civil societies involved in providing care services.

#### 3.2.2 Countries provide diagnostic guidelines to support GPs and other health professionals

Some countries have taken steps to improve the quality of dementia diagnosis through the development of guidelines. Diagnostic guidelines are currently available in 30 OECD countries (Table S2 in supporting information), with some countries having implemented more than one (e.g., Australia, Canada, Chile, Germany, Latvia, Mexico, Switzerland, or the US).

Among the 38 dementia diagnostic guidelines available across 30 OECD countries, there is considerable variation in how often they are updated, their scope, target audiences, and how they assess evidence quality. Sixty‐five percent of these guidelines (25/38) have been updated since 2018. Regular updates ensure guidelines reflect the latest evidence, medical advancements, and evolving best practices. In the US, diagnostic criteria were updated to reflect new science and technologies, such as the use of plasma biomarkers, alongside validated cerebrospinal fluid and imaging markers from the 2018 framework. In contrast, eight countries (Belgium, Greece, Lithuania, Luxembourg, Poland, Portugal, Slovak Republic, and Slovenia) did not have a guideline in place as of September 2025. Costa Rica relies on national legislation to address many functions usually covered by a dementia diagnosis guideline (National Norm for Care of Adults with Cognitive Impairment and Dementia from 2017). Czechia is in the process of developing a proposal for indications for diagnosis and treatment with relevant professional health insurance companies.

Guidelines support GPs and other health and social workers who may lack specific dementia training by incorporating standardized diagnostic tools, subtype‐specific diagnostic criteria, principles of communication and care, ethical considerations, and post‐diagnostic care pathways. They contribute to standardizing diagnostic processes, ensuring consistency and timeliness in clinical decision making. Most dementia guidelines across OECD countries recommend a similar set of diagnostic procedures. The diagnostic process typically follows a structured pathway initiated by a referral from a GP. This pathway generally includes a clinical interview with the patient (often involving a family member), a physical and neurological examination, cognitive testing, laboratory tests (such as blood work), and neuroimaging techniques (like magnetic resonance imaging or computed tomography scans) to assess brain structure and function.

Primary care doctors use a range of standardized tools and tests to assess people with suspected dementia, which are often recommended in clinical guidelines. Among diagnostic guidelines, 24 countries recommend the Mini‐Mental State Examination, with 12 of them recommending it alongside the Clock Drawing Test. The Montreal Cognitive Assessment test is recommended by 20 countries for detecting mild cognitive decline, as it is considered more accurate in early stages.

Guidelines serve as a useful tool for GPs to deliver more consistent, evidence‐based care tailored to dementia patients. Some guidelines grade the quality of evidence and strength of recommendations, such as Australia, Denmark, Germany, Hungary, Iceland, Italy, Japan, Korea, and Latvia. For example, Germany uses the GRADE approach, a four‐level system that rates the quality of evidence as high, moderate, low, or very low. In addition, they include two‐stage recommendation grading: a strong recommendation is expressed as “we recommend” or “do not recommend” (⇑⇑/⇓⇓), while a weaker recommendation is expressed as “we suggest” or “do not suggest” (⇑/⇓). Similarly, Australia follows the GRADE approach, while recommendations are classed differently as “evidence‐based recommendations” developed from systematic reviews with supporting references; “consensus‐based recommendations” issued when no quality evidence is available despite a systematic review; or “practice points,” which offer guidance outside the scope of the evidence review. Thanks to this, users can trust the evaluated information and whether adherence to the recommendations will yield more benefits than harm. Moreover, access to this information is useful for the other health and social care professionals supporting GPs in the diagnosis, leading to earlier diagnosis and timely intervention.

### 3.3 Treatment

#### 3.3.1 Clinical guidelines for treating dementia have evolved over the past decade

Countries have established clinical guidelines for dementia care to provide standardized recommendations based on evidence and ensure quality care throughout the progression of the illness across all care settings. Currently, at least 36 clinical guidelines for dementia care are available across 27 countries (Table 2).

**TABLE 2:** Overview of focus areas across guidelines for dementia treatment in 27 OECD countries.

Country	Year	Pharmacological approaches	Non‐pharmacological interventions	Care pathways	Support for caregivers and families	Hospital and acute care management	Ethical and legal considerations
Australia	2015/2016	Medium	High	High	High	High	Medium
Austria	2011	Medium	Medium	Medium	Medium	Low	Low
Canada	2020	Medium	High	Medium	Medium	Low	Low
Denmark	2018	High	Medium	Medium	Medium	Low	Medium
Estonia	2017	Medium	Medium	Medium	Medium	Low	Low
Finland	2024	High	High	High	High	Low	High
France	2018	Medium	High	High	High	Medium	High
Germany	2023	High	High	Medium	Medium	Medium	Medium
Hungary	2022	Medium	Medium	Medium	Medium	Low	Medium
Iceland	2007	Medium	Medium	Medium	Medium	Low	Medium
Ireland	2023	Medium	High	High	High	High	Medium
Israel	2023	Medium	Medium	Medium	Medium	Low	Low
Italy	2024	High	High	High	Medium	Medium	Medium
Japan	2017	Medium	Medium	Medium	Medium	Low	Medium
Korea	2022	Medium	Medium	Medium	Low	Low	Low
Latvia	2017	High	Medium	Medium	High	Low	Low
Mexico	2017	Medium	Low	Low	Medium	Low	Low
Netherlands	2024	Medium	High	High	High	Medium	Medium
Norway	2024	Medium	High	High	High	Medium	High
Portugal	2023	Medium	Medium	Medium	Medium	Low	High
Scotland	2023	Medium	High	High	High	Medium	Medium
Slovenia	2013	Medium	Medium	Medium	Medium	Low	Medium
Spain	2018	Medium	Medium	Medium	Medium	Low	Low
Sweden	2017	Medium	High	High	High	Medium	Medium
Switzerland	2018	Medium	Medium	Medium	Medium	Low	High
United Kingdom	2018	Medium	High	High	High	Medium	Medium
United States	2018	Medium	High	High	High	Medium	Medium

*Note*: Each treatment guideline is evaluated based on the level of detail and tone of each recurring theme. “High” indicates that the guideline provides a comprehensive and detailed discussion of the respective theme with a strong emphasis, “Medium” signifies a broad discussion with a moderate emphasis, and “Low” denotes that the theme is either briefly discussed without details, with relatively low or no emphasis, or not addressed at all. *Source*: OECD own analysis.

Abbreviation: OECD, Organisation for Economic Co‐operation and Development.

Twenty out of twenty‐seven countries (74%) have nationally applicable care guidelines that are either published or endorsed by the government, one country (4%) has a subnational level guideline, and nine countries (33%) have sector‐specific guidelines published by relevant medical associations or dementia‐oriented non‐governmental organizations (overlaps permitting). Australia, Estonia, France, Ireland, Japan, and Sweden have more than one guideline by different authorities. Most of the guidelines identified were released in 2018 or later, while the national treatment guidelines from Australia, Austria, Estonia, Iceland, Japan, Mexico, Slovenia, and Sweden have not been updated between 2018 and September 2025. While Czechia and Estonia are developing clinical treatment guidelines, Costa Rica, Greece, and Poland lack a single dementia‐specific guideline.

These clinical guidelines for dementia commonly address non‐pharmacological interventions, pharmacological approaches, care pathways or models of care, support for caregivers and families, management of hospital and acute care, ethical and legal considerations, along with public health and prevention, and diagnosis and assessment. Non‐pharmacological interventions are among the most frequently addressed (29 out of 33), whereas ethical and legal considerations were the least (20 out of 33).

#### 3.3.2 Non‐pharmacological interventions are considered the first‐line treatment

The treatment guidelines identified recommend 14 types of non‐pharmacological interventions. Included are cognitive stimulation therapy, reminiscence therapy, music therapy, physical activity or exercise, occupational therapy, environmental modifications, validation therapy, psychosocial interventions, caregiver support and education, structured routines, person‐centered care, multicomponent interventions, assistive technology, and social engagement activities. In practice, physical exercise was the most used non‐pharmacological intervention, followed by cognitive stimulation therapy, psychological therapy, and social activities, according to the 2024 OECD dementia interviews and questionnaire.

Cognitive stimulation therapy, a series of individual or group sessions used to help strengthen a person's communication skills, thinking, and memory through games, singing, and discussion, is now more widely recognized as a core non‐pharmacological intervention for people with mild to moderate dementia than in 2018, when it was recognized as promising but not universally adopted.^4^ Physical exercise has also gained increasing support in guidelines in terms of both the prevention and management of dementia, although the scientific certainty remains low to moderate, especially regarding slowing progression from mild cognitive impairment to dementia.^17^ Guidelines emphasize implementation based on holistic health benefits, even if cognitive outcomes are not definitively proven. Specifically, multicomponent interventions (combining physical, cognitive, and social activities) are emphasized in newer guidelines as deemed to be more effective (e.g., Germany and Australia). In several countries, ongoing non‐pharmacological interventions alongside pharmacological treatment are recommended (e.g., Canada, England, and Scotland).

In addition, countries are making efforts to improve the environment for managing dementia in hospitals and the community. Current guidelines (e.g., Australia's Safety and Quality Pathway, Ireland's Integrated Care Pathways) include hospital‐specific protocols for managing delirium and dementia, and promote dementia‐friendly environments, as hospitals were identified as high‐risk environments for people with dementia in 2018.^4^ Meanwhile, the significant care burden and lack of structured support have been recognized as ongoing issues. Most guidelines now include formal caregiver support, such as education, respite care, and psychological services (e.g., NICE NG97, Alzheimer's Association recommendations).

#### 3.3.3 Pharmacological treatment is recommended as second‐line treatment

Four dementia medications currently available—memantine, donepezil, galantamine, and rivastgmine—are reported to have moderate effects on cognition for people with different types of dementia^18^, ^19^ with some considerable side effects.^20^ With limited treatment options, pharmacological interventions are widely seen as second line after non‐pharmacological care, though national practices vary. Australia, Norway, and the UK take cautious, evidence‐based approaches, emphasizing risk–benefit assessment, safety, and person‐centered care, particularly for older adults. In contrast, 10 countries (Denmark, France, Germany, Hungary, Israel, Italy, Japan, Korea, Mexico, and Portugal) use structured, specialist‐led models in which medication is central; Germany provides detailed protocols, while Korea stresses neurologist and psychiatrist oversight. Nine countries (Austria, Canada, Estonia, Finland, Ireland, Netherlands, Scotland, Switzerland, and the US) favor integrated, person‐centered care that combines medication with psychosocial support, caregiver education, and environmental adaptations. Overall, these approaches reflect differing priorities, from safety and regulation to clinical precision and holistic care.

## 4 DISCUSSION

Recent evidence from the Lancet Commission has revealed new evidence on 14 modifiable risk factors, indicating their potential to prevent or significantly delay ≈ 45% of all dementia cases.^21^ The latest dementia care policies increasingly recognize prevention as a central pillar, with growing evidence that a substantial proportion of risk can be reduced through population‐level interventions across the life course. With declines in smoking, countries have made preventive efforts to make mental health treatment more accessible and promote physical exercise among people aged ≥ 65. The incidence rate of dementia in Europe and North America has declined by 13% per decade over the past 25 years, which could lead to 15 million fewer people developing dementia by 2040.^22^ This shifts dementia from a solely clinical challenge to a public health priority, requiring coordinated action throughout health systems.

### 4.1 With the dementia policy frameworks established, countries’ levels of commitment and implementation vary

Across the OECD, dementia care policies are currently guided by structured national strategies, diagnostic guidelines, and treatment guidelines that allow countries to define objectives and to measure progress against these targets. Most countries currently have a stand‐alone or integrated dementia plan as part of a broader strategy on aging or brain health, as well as clinical guidelines for diagnosis and treatment. However, progress in updating and establishing national dementia strategies has been uneven across OECD countries since 2018.

Beyond the common themes identified across national strategies, the priority areas may also vary across countries. Community‐based services are essential as they provide accessible care and ensure continuity across various stages of dementia. In Canada and Costa Rica, dementia strategies include an objective on the importance of care coordination and strengthening community‐based care services. Similarly, in Korea and Japan, both national dementia plans include a network of local dementia centers to provide community‐based diagnostic and care.^23^

We note that not all strategies are accompanied with designated funding allocations or monitoring and evaluation plans that ensure they are translated into tangible changes.^24^ Denmark is an exception that has recently implemented initiatives to promote early diagnosis of dementia. As part of the 2025 Finance Act, Denmark has allocated DKK 15 million for 2025 and DKK 20 million for 2026 to strengthen regional dementia assessments by expanding capacity and reducing waiting times. This reflects Denmark's ongoing commitment, as shown by the 2024 Health Agreement (Sundhedsaftale), which earmarked over DKK 123 million to upgrade assessment units, upskill staff, and support safe prescribing, including antipsychotics.^25^

### 4.2 Support for diagnostic capacity among health‐care professionals could be further prioritized to encourage early detection of dementia

While countries have established diagnostic guidelines that support the health workforce in better detecting dementia symptoms, significant issues still hamper a strong uptake of dementia diagnosis to close the diagnostic gap between estimated prevalence and confirmed diagnoses. Several OECD countries have seen an increase in dementia diagnoses in absolute terms, while the aging population also drives up the demand for diagnoses. A shortage of GPs, as reported in Austria, Finland, Italy, Latvia, Portugal, Slovenia, and Spain,^26^ and bottlenecks in appropriate and accurate diagnosis can also contribute to delays and inequalities in access to specialists and diagnostic equipment.

Over the years, several diagnostic tests have become available, including blood‐based biomarkers. Blood‐based biomarkers are promising predictive tools, and paired with the availability of new pharmacological treatments, they offer new hope for better, earlier treatment of dementia for some patients.^27^ The new predictive tools are less invasive than existing diagnostic methods (such as cerebrospinal fluid) and require less advanced technology, which makes them easier to access, cheaper, and scalable. With improved diagnosis accuracy of biomarkers, it is estimated that this could reduce specialist evaluations from 36% to 14%, thereby saving costs substantially by performing diagnosis at the primary care setting.^28^

That said, the new technology is in the development phase, and countries may need to prioritize strengthening the diagnostic capacity within the health workforce. Our review finds that only a few OECD countries have expanded the types of health professionals authorized to diagnose dementia to increase the capacity of health workers to make diagnoses. In general, GPs remain the main point of contact for dementia diagnosis in many countries. However, people suspected of having dementia often experience limited consultation time and with other acute and chronic diseases that need to be taken care of during a consultation with their GP.

### 4.3 Non‐pharmacological approaches need systematic support to be integrated into standard dementia care practices

The latest treatment guidelines have expanded non‐pharmacological therapies while underscoring the cautious use of pharmacological treatments. Guidelines from Australia, Canada, and the UK now prioritize non‐pharmacological approaches such as cognitive stimulation, music therapy, and environmental design. Together, many countries (e.g., Denmark, Germany, and Ireland) have updated their guidelines to limit antipsychotic use, promote deprescribing, and emphasize non‐drug alternatives for behavioral symptoms.

The recent pharmaceutical innovations (lecanemab and donanemab) have brought new hope to dementia treatment but also present new challenges. The new drugs are designed for early‐stage AD (mild cognitive impairment or mild dementia), aiming to slow disease progression by clearing amyloid plaque in the brain.^29^ They differ from the existing drugs that are used across mild to moderate stages of AD, primarily to manage symptoms. Despite potential benefits, a significant share of people in clinical trials reportedly experienced mild brain swelling and bleeding side effects.^30^ Currently, not all OECD countries have approved the new medications and there is controversy about the cost–benefit of the new medications beyond their effectiveness. More R&D for new treatments that provide a cure rather than reducing the symptoms remain necessary.

With currently limited pharmacological options to treat dementia, non‐pharmacological approaches have become the standard and preferred interventions for improving the quality of life for persons with mild cognitive symptoms, along with their caregivers.^4^ Compared to a decade ago, increasing evidence indicates that non‐pharmacological interventions are effective in supporting or improving cognitive function in people with mild to moderate dementia, although the degree of benefit varies by intervention type and individual factors.^31^ Despite growing attention and increasing evidence, they are not yet widely adopted in clinical practice due to limited awareness among physicians and families and insufficient reimbursement rates.

## 5 CONCLUSIONS

Dementia has become major topic in public health, corresponding to the growing number of people affected and the costs incurred by the disease. We report on recent trends in dementia care policy in the OECD by reviewing the latest policy documents from 38 OECD member countries, focusing on national dementia strategies, diagnostics, and treatments. Almost all countries studied have national dementia strategies and clinical guidelines, all of which have become more comprehensive and diverse in care options, reflecting the complexity of dementia. Still, we identify a gap in translating policy into action to overcome diagnostic bottlenecks and improve care for people living with dementia, while improvements in care risk merely keeping up with increased demand due to aging. Our findings fill a gap in policy research by providing a comprehensive overview of recent changes in dementia care policies across all OECD countries, regardless of languages of origin.

## CONFLICT OF INTEREST STATEMENT

The authors declare no conflicts of interest. Author disclosures are available in the Supporting Information.

## Supporting information



Supporting Information

Supporting Information

Supporting Information
