# Supplementary material for: Evolving national dementia policies in the OECD: Prevention, diagnosis, and care
Source: Alzheimers Dement. 2026 Jun 1;22(6):e71367. doi: 10.1002/alz.71367 (PMC13239349; doi:10.1002/alz.71367)
Supplement: Supplementary file 1 — Supporting Information [file ALZ-22-e71367-s001.docx]

**Table A1. Across OECD countries, while GPs are the main contact point, specialists perform the formal diagnosis**

|  | **2018** | **2025** |
| --- | --- | --- |
| **GPs as the first contact point** | Australia, Austria, Belgium, Canada (British Columbia), Chile, Czechia, Denmark, Estonia, Finland, France, Germany, Hungary, Ireland, Israel, Luxembourg, Mexico, Netherlands, New Zealand, Norway, Portugal, Slovak Republic, Slovenia, Sweden, Switzerland, United Kingdom (England), United States. | Australia, Austria, Belgium, Canada, Chile, Colombia, Costa Rica, Czechia, Denmark Estonia, Germany, Greece, Finland, France, Hungary, Ireland, Iceland, Israel, Italy, Japan, Korea, Latvia, Lithuania, Luxembourg, Mexico, the Netherlands, New Zealand, Norway, Poland, Portugal, Slovenia, Slovak Republic, Spain, Sweden, Switzerland, Türkiye, United Kingdom, United States. |
|  | **2018** | **2025** |
| **Specialists performing a formal diagnosis** | Austria, Belgium Czechia, Estonia Finland, France, Germany, Greece, Hungary, Ireland, Israel, Luxembourg, Mexico Portugal, Slovak Republic, Slovenia, United States. | Australia, Austria, Belgium, Chile, Colombia, Costa Rica, Czechia, Denmark Estonia, Germany, Greece, France, Finland, Hungary, Iceland, Ireland, Israel, Italy, Japan, Korea, Latvia, Lithuania, Luxembourg, Mexico, the Netherlands, New Zealand, Norway Poland, Portugal, Slovenia, Slovak Republic, Spain, Sweden, Switzerland, Türkiye, United Kingdom, United States. |
