# Supplementary material for: Evolving national dementia policies in the OECD: Prevention, diagnosis, and care
Source: Alzheimers Dement. 2026 Jun 1;22(6):e71367. doi: 10.1002/alz.71367 (PMC13239349; doi:10.1002/alz.71367)
Supplement: Supplementary file 2 — Supporting Information [file ALZ-22-e71367-s002.docx]

**Table A2. Countries have made efforts to promote early dementia detection through the implementation of diagnostic guidelines**

| **Countries** | **Name of Policy** | **Year** | **Audience** |
| --- | --- | --- | --- |
| Australia | Clinical Practice Guidelines and Principles of Care for people with dementia | 2016 | Health and aged care staff: doctors, nurses, allied health and care workers (community, residential, hospital), people with dementia & carers |
|  | Guidelines for Diagnosis and Care of Aboriginal People with Dementia in Remote Communities | 2012 | Health professionals and aged care coordinators. |
| Austria | Medical Guideline - Living Better with Dementia | 2019 | Primary care and specialist physicians |
| Canada | Recommendations of the 5th Canadian Consensus Conference on the diagnosis and treatment of dementia | 2020 | Clinicians and researchers |
|  | National Dementia Guidelines: Disclosing and Communicating a Diagnosis of Dementia | 2024 | Family physicians and health care providers/professionals |
| Chile | Clinical guidelines for the diagnosis and treatment of dementias | 2017 | Not specified |
|  | Technical guidance for the implementation of Explicit Health Guarantees (GES) No. 85 for Alzheimer’s and other dementias | 2022 | Primary and specialized healthcare teams for the care of individuals with dementia and their surrounding environment |
| Colombia | Clinical practice guideline for the diagnosis and treatment of major neurocognitive disorder (dementia) in Colombia | 2017 | Healthcare professionals |
| Costa Rica | National Norm for Care of Adults with Cognitive Impairment and Dementia | 2017 | Not specified |
| Czechia | Recommendations for the Diagnosis and Management of Alzheimer’s Disease and Other disorders associated with dementia | 2008 | Neurologists, geriatricians, psychiatrists and other specialised physicians responsible for the care of patients with dementia |
| Denmark | National clinical guideline for diagnosis of mild cognitive impairment and dementia | 2017-2020. | Not specified |
| Estonia | Alzheimer’s Disease Diagnosis and Treatment | 2017 | Healthcare professionals: family physicians, specialists, clinical psychologists, speech therapists, occupational therapists, nurses, etc. |
| Finland | Memory Disorders Current Care Guidelines | 2023 | Doctors, healthcare professionals, social workers, pharmacists, students, decision-makers in social welfare and healthcare, and, where applicable, memory patients and their families |
| France | Guide care pathway for Alzheimer's disease or a related disorder. | 2018 | Health professionals |
| Germany | S3 living guideline on dementia | 2025 | Specialist doctors, neuropsychologists, occupational therapists, physiotherapists, art therapists, music and dance therapists, speech therapists, nursing staff, social workers, affected individuals & relatives |
|  | S2k Guideline: Consent of People with Dementia to Medical Measures | 2019 | Doctors, psychologists, and nurses from participating professional associations and organisations serve to inform other individuals (e.g., doctors from other professional associations, other therapeutic professions, and social workers) |
| Hungary | Diagnosis, Treatment, and Care of Dementia | 2022 | Physicians, psychologists, physiotherapists, dieticians, nursing staff, caregivers |
| Iceland | Dementia. Diagnosis and treatment | 2007 | Healthcare professionals |
| Ireland | Dementia Diagnosis & Management in General Practice: | 2019 | GPs, public health nurses, occupational therapists, physiotherapists, speech language therapists, dieticians, social workers |
| Israel | Dementia treatment and its prevention | 2022 | Physicians |
| Italy | National guideline “Diagnosis and treatment of dementia and Mild Cognitive Impairment” | 2024 | All health and social care professionals involved in caring for people with dementia or MCI in any setting |
| Japan | Clinical Practice Guideline for Dementia | 2017 | Doctors, but also other professionals |
| Korea | Dementia Clinical Treatment Guidelines. | 2021 | Psychiatrists and neurologists, as well as internists, family medicine doctors, and primary care physicians who may encounter dementia patients |
| Latvia | Clinical pathways and algorithms: “Diagnosis and Treatment of Dementia” | 2019 | GPs, psychiatrists, neurologists, other specialised doctors, specialists from diagnostic offices, patients and their relatives, support persons |
|  | Clinical guidelines for Alzheimer's disease, vascular dementia, Lewy body dementia, and frontotemporal dementia | 2017 | Neurologists, psychiatrists, internists, family (general practice) doctors, residents of the relevant specialties, and medical faculty students for training purposes |
| Mexico | Diagnosis and Treatment of Dementia in Older Adults at the Primary Care Level | 2009 | General practitioners, family doctors and psychologists |
|  | Updated for vascular dementia for 3 care levels | 2017 | Specialist Doctors, General Practitioners, Family Doctors, students |
| New Zealand | Guidance on recognising and managing early dementia | 2020 | Not specified |
| Norway | National professional guideline | 2022 | Primary and secondary healthcare professionals, social care professionals, community, care home and care at home staff, people living with dementia and their families and carers |
| Scotland (UK) | Assessment, diagnosis, care and support for people with dementia and their carers | 2023 | Doctors, especially neurologists, psychiatrists, geriatricians, internists and family doctors and other healthcare professionals |
| Spain | Diagnostic and therapeutic guidelines of the Spanish Society of Neurology | 2018 | Not specified |
| Sweden | National guidelines for care in dementia | 2018 | Patients |
| Switzerland | Medical Ethical Guidelines. Care and treatment of people with dementia. | 2017 | Physicians, nurses and therapists caring for patients with a persistent disease-related loss of cognitive abilities |
|  | Diagnostic recommendations for dementia by Swiss Memory Clinics | 2024 | Professionals in primary care and memory clinics |
| The Netherlands | Dementia Diagnostics | 2024 | All care providers involved in the secondary and third line care of patients with dementia or suspected dementia |
| Türkiye | Clinical protocol for Alzheimer's and other dementia diseases | 2025 | All physicians involved in the diagnosis and treatment of diseases causing dementia, especially Alzheimer’s disease |
| United States | Physician Guidelines for the Screening, Evaluation, and Management of Alzheimer’s Disease and Related Dementias - San Diego Region | 2024 | Primary care physicians, internists, psychiatrists, nurse practitioners, and physician assistants caring for older adults in their practices |
|  | Revised Criteria for Diagnosis and Staging of Alzheimer's Disease: Alzheimer’s Association Workgroup | 2024 | Not specified |
